# Structure-guided analysis of divergent homologs unveils deep ancestry and arthropod specialization of the pacifastin family

**DOI:** 10.1038/s41598-026-52748-5

**Published:** 2026-05-20

**Authors:** Eduardo D. Rodríguez-Aguilar, Jesús Martínez-Barnetche, Mario H. Rodríguez

**Affiliations:** Centro de Investigación Sobre Enfermedades Infecciosas, Instituto Nacional de Salud Pública, Av. Universidad 655, C.P. 62100 Cuernavaca, Morelos Mexico

**Keywords:** Structural phylogenomics, Serine protease inhibitors, Structure-based homology, Protein architecture, Molecular diversification, Biochemistry, Computational biology and bioinformatics, Evolution, Structural biology

## Abstract

The Pacifastin family has been widely regarded as an arthropod-specific innovation, functionally restricted to the regulation of innate immunity. Here, we challenge this paradigm using a structural phylogenomics approach that overcomes the sequence erosion characteristic of small disulfide-rich proteins. We identified a population of “cryptic” homologues comprising ~ 30% of the dataset—undetectable by sequence alone and demonstrated that the characteristic Pacifastin’s six-cysteine scaffold is not an arthropod invention but an ancient lineage whose evolutionary roots extend across major metazoan phyla, Fungi, and Bacteria. Structural and architectural profiling revealed two recurrent organizational modes: an ancestral class characterized by an N-terminal β-hairpin extension, glycine-rich and frequently embedded in multidomain, extracellular matrix–associated proteins, and a conventional, derived arthropod-restricted class characterized by modular simplification and predicted structural rigidity consistent with a soluble-effector function. We propose a "Liberation and Structural Convergence" evolutionary model to explain this transition: the acquisition of proteolytic processing sites in inter-domain linkers is consistent with a mechanism by which arthropods may have released these domains from the matrix, while the concurrent structural convergence is consistent with adaptation to a soluble, circulating hemolymph environment. Further phylogenomic profiling of the reactive site revealed a functional shift from an ancestral inhibitory signature enriched in basic and charged residues to a derived arthropod-specific radiation toward hydrophobic residues. This transition suggests a broadening of target specificity toward chymotrypsin- and elastase-like proteases, consistent with the recruitment of these inhibitors into arthropod immune roles. Together, these results reposition pacifastins as an ancient protein lineage and illustrate how modularity, proteolytic processing, and biophysical constraints may have driven the transition from matrix-embedded regulatory scaffolds to systemic soluble effectors.

**Supplementary Information:**

The online version contains supplementary material available at 10.1038/s41598-026-52748-5.

## Introduction

Serine protease inhibitors (SPIs) are pivotal regulators of a broad spectrum of physiological and cellular functions, modulating proteolytic cascades with crucial roles in metabolism, protein folding, hemostasis, immune response, cell migration, and extracellular matrix reconstruction^1–4^. These inhibitors function as essential components of the innate immune system, neutralizing virulence factors secreted by pathogenic microorganisms and regulating endogenous signaling pathways^5,6^. While major families such as Kunitz, Kazal, and Serpins have been extensively characterized^7–10^, other cysteine-rich inhibitor families remain less understood, potentially harboring unexplored evolutionary histories and functional adaptations.

Among these, the Pacifastin family represents a unique class of inhibitors. First described in the late 90s in the plasma of the freshwater crayfish *Pacifastacus leniusculus*^11^, pacifastins are characterized by a distinctive structural signature. The functional unit, the Pacifastin-like domain (PLD), consists of approximately 35 amino acid residues stabilized by a strictly conserved pattern of six cysteines that form three specific disulfide bridges (*Cys1–Cys4, Cys2–Cys6, Cys3–Cys5*)^12^. This arrangement confers a stable folding that supports an exposed canonical binding loop containing the reactive site (P1 residue)^13,14^. Pacifastins were identified primarily as large multi-domain precursors containing frequent dibasic cleavage sites between domains, suggesting a mechanism for the post-translational release of single- or multi-domain inhibitory peptides^15^. Biochemical characterization showed that their specificity is determined by amino acid residues in the P1 position: variants with Lysine or Arginine inhibit trypsin, while those with Phenylalanine or Leucine target chymotrypsin-like proteases^16^.

Despite their structural robustness, the evolutionary history of pacifastins has been obscured by taxonomic bias. Previous in silico analyses led to the assumption that the family was restricted to the Arthropoda phylum, specifically within insects and crustaceans^17,18^. A previous large-scale in silico survey provided the first evidence that the family extended beyond Arthropoda, identifying a total of 83 pacifastin homologs containing 284 inhibitor domains, which were distributed across 55 species from 3 metazoan phyla. Non-arthropod single representation each were iidentified in Placozoa and in Onychophora (4 PLDs)^19^. However, the isolated presence of pacifastins in basal lineages raises the question of whether their apparent restriction to Arthropoda is biological or the result of methodological limitations. Since small, disulfide-rich proteins evolve rapidly^20,21^, probably leading to extreme sequence divergence that erodes the phylogenetic signal detectable by standard homology search tools (e.g., BLAST, HMMER)^22,23^, we hypothesized that the registered distribution represents only a fraction of their true diversity. Consequently, a significant portion of the pacifastin evolutionary tree may exist as “cryptic” homologues (*i.e., sequences undetectable by profile-based sequence searches despite possessing the Pacifastin structural fold — hereafter referred to as ‘cryptic’ in this operational sense, independently of their structural classification*).

To overcome the limitations of sequence-based detection, we implemented a structural phylogenomic approach. By leveraging recent advances in protein structure prediction (AlphaFold2/ESMFold)^24^ and high-throughput structural alignment (Foldseek)^25^, we systematically screened the proteomes of diverse metazoan and non-metazoan lineages. We assumed that the persistence of the six-cysteine structural scaffold would allow us to trace the lineage back to its true origins, bypassing the limitations of primary sequence divergence.

In this study, we redefine the Pacifastin family, demonstrating that the Pacifastin-like Domain (PLD) scaffold did not originate within the Arthropoda; rather, it represents an ancient lineage with deep roots extending to Fungi, Rotifera, and Cnidaria. Consequently, the isolated homologues previously identified in Placozoa and Onychophora are not evolutionary anomalies, but integral members of a broad, continuous evolutionary history that ultimately gave rise to the specialized inhibitors found in modern arthropods. We identified two distinct architectural lineages: a “Conventional” form restricted to arthropods and a novel “Ancestral” architecture that dominates basal lineages. In addition, by integrating structural landscape analysis with cleavage site profiling, we propose a new evolutionary model, where the acquisition of proteolytic processing sites in arthropods is statistically associated with the transition from ancient, flexible, matrix-tethered scaffolds to the rigid, soluble, and highly specialized immune effectors found in modern species, consistent with a selective process driving this architectural shift.

## Results

### Identification of cryptic pacifastins and phylogenetic distribution

**Fig. 1:**
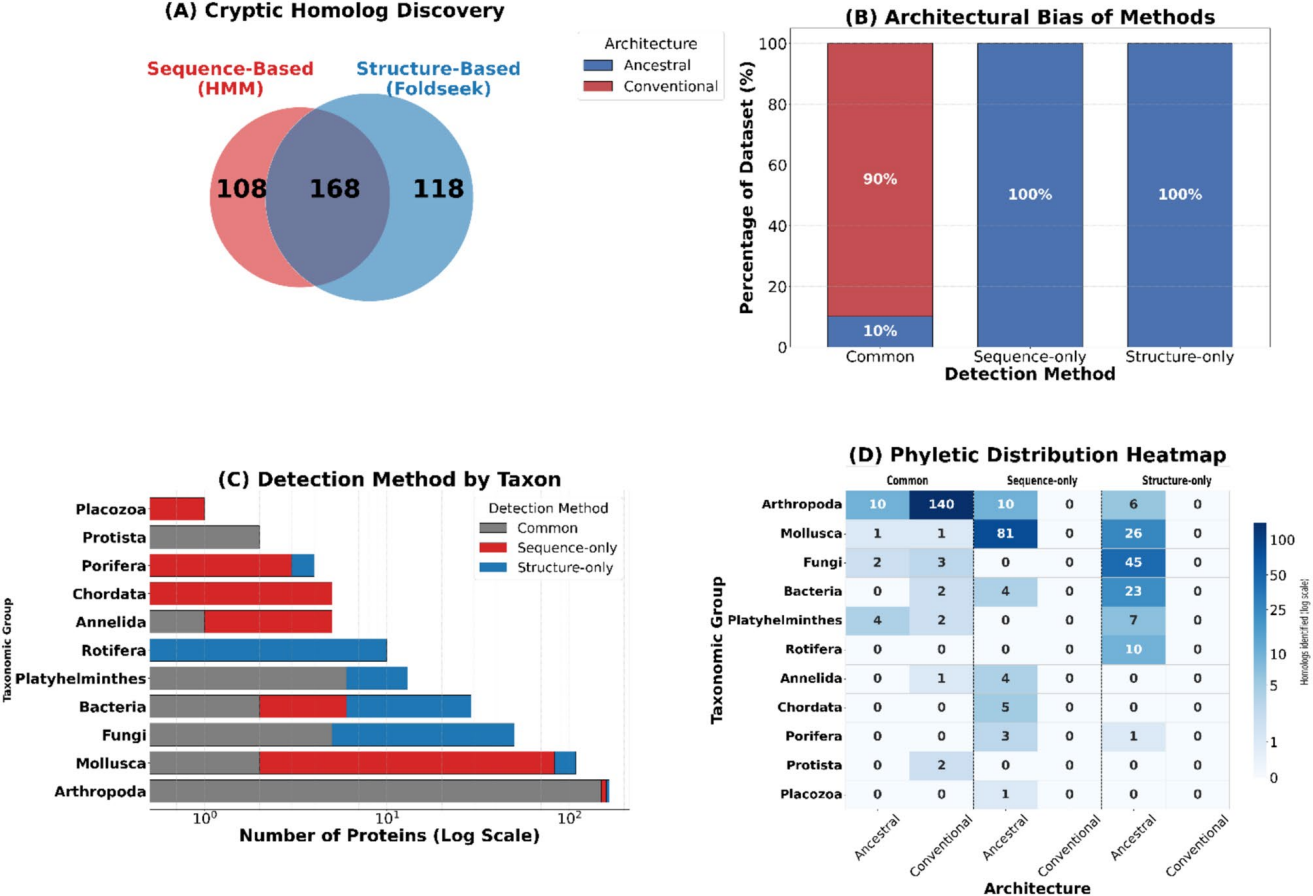
Discovery of a cryptic Pacifastin proteome. **(A)** Venn diagram illustrating the comparative sensitivity of sequence-based (HMM) versus structure-based (Foldseek) detection strategies within the validated dataset (n = 394). Structural search revealed a distinct population of 118 “cryptic” proteins (29.9%, Structure-only) undetectable by sequence profiles. **(B)** Architectural bias of detection methods. The “Common” dataset is dominated by the Conventional (reduced N-terminal region) architecture, while the Structure-unique dataset is composed exclusively of the Ancestral (N-terminal β-hairpin extension) architecture, indicating that structural divergence in the N-terminal region correlates with the loss of sequence identity. **(C)** Stratification of detection methods by Taxonomic Group (logarithmic scale). The bar chart reveals a strong methodological bias: Arthropods are well-represented by sequence-based methods (Gray/Red bars). In contrast, distant groups such as Fungi and Rotifera were identified predominantly or exclusively via the structure-based approach (Blue bars). Notably, Rotifera represents a lineage entirely invisible to sequence-based search methods. **(D)** Phyletic distribution heatmap. Number of homologs identified per taxonomic group (rows) stratified by detection method (Common, Sequence-only, Structure-only) and architectural class (Ancestral, Conventional) (columns). Color intensity is proportional to the number of sequences on a log scale; cell annotations indicate raw counts.

A comprehensive iterative search for Pacifastin-like Domains (PLDs) using both sequence-based (HMM) and structure-based (Foldseek) profiling followed by manual curation to validate the canonical six-cysteine core and redundancy filtering, established a final high-confidence dataset of 394 unique protein sequences containing at least one confirmed PLD. A comparative analysis of the detection methods revealed a critical methodological divergence. While 168 proteins (42.6%) were consistently detected by both strategies (“Common core”), a significant subset of 118 proteins (29.9%) was identified exclusively through structural alignment (Fig. 1A). These “cryptic” homologues possess the canonical Pacifastin fold but diverged sequence beyond the detection limit of sensitive HMM profiles (E-value > 0.01). Conversely, 108 proteins (27.4%) were detected only by sequence, primarily representing highly divergent sequences that failed to meet the strict TM-score structural threshold, despite retaining key sequence motifs.

This cryptic population corresponds to a distinct, previously uncharacterized structural lineage. A fundamental bifurcation in the PLD topology was observed based on the N-terminal region : a “Conventional” architecture (canonical short loop, 9–12 residues) and a novel architecture characterized by an N-terminal β-hairpin extension of 13 to 20 residues. We designated this latter group as “Ancestral” architecture, as subsequent phylogenomic and evolutionary metric analyses (detailed in later sections) identified it as the plesiomorphic state of the family. As shown in Fig. 1B, this architectural divergence is the main driver of detection bias. The “Common” dataset is dominated by conventional architecture (90%). In contrast, the "Structure-only" dataset is composed exclusively of the Ancestral architecture (100%).

**Fig. 2:**
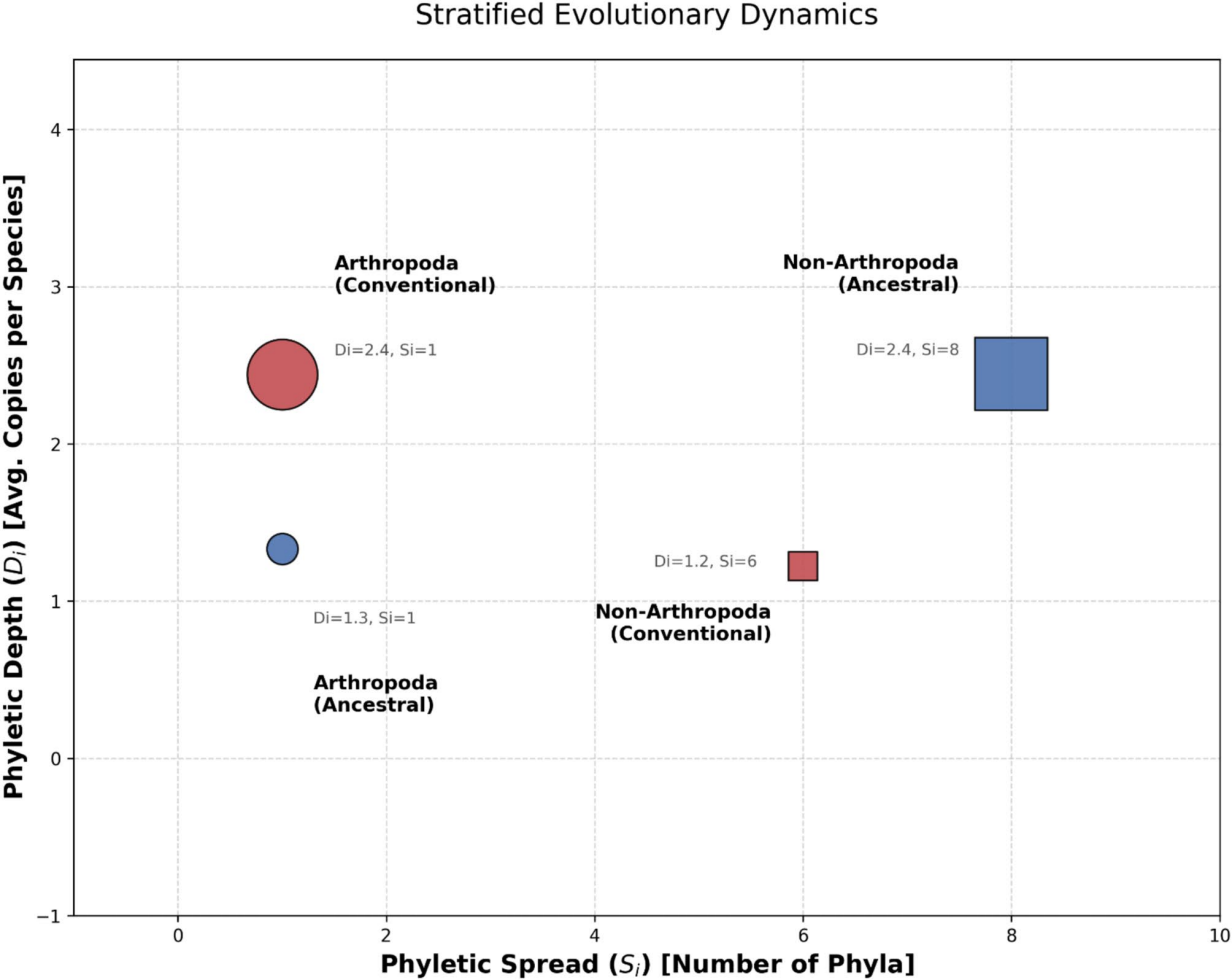
Phyletic Spread (Si) and Phyletic Depth (Di) for Pacifastin lineages stratified by taxonomic group. *Si* is the number of unique phyla/kingdoms in which a lineage is present; *Di* is the average gene copy number per species. Data points are stratified by major taxonomic grouping (Arthropoda vs. Non-Arthropoda) to resolve lineage-specific trends. The size of markers is proportional to the total number of sequences analyzed (*n*). Within Arthropoda, both lineages are restricted to a single phylum (*Si* = 1) but diverge markedly in copy number: the Conventional lineage shows higher depth (*Di* = 2.4) than the Ancestral form (*Di* = 1.3). Outside Arthropoda, the Ancestral lineage displays the broadest taxonomic distribution (*Si* = 8, *Di* = 2.4), spanning basal metazoans, Lophotrochozoa, Fungi, and Bacteria, while the Conventional lineage shows more restricted spread (*Si* = 6, *Di* = 1.2).

To determine the evolutionary origin of this lineage, we stratified the detection methods by taxonomic group (Fig. 1C). The analysis revealed that the Ancestral architecture was not merely a rare variant, but the basal state of the family. Arthropoda, which dominates the dataset (n = 166), was the only taxa characterized by the conventional architecture and was robustly detected by sequence-based methods, with 90.3% of its members found in the Common or Sequence-only sets. In contrast, homologues identified in distant deep-branching taxa were detected predominantly via the structure-based approach. Notably, Rotifera (n = 10) was identified exclusively by structural homology, with zero sequence-based hits. Similarly, Fungi showed a massive cryptic population, with 45 structure-unique proteins compared to zero sequence-unique hits, and Bacteria displayed a high proportion of structural hits (23 structure-unique). Mollusca, the second largest group (n = 109), exhibited a mixed profile, retaining a significant number of sequence-identifiable homologues (81) alongside a substantial cryptic population (26). Proteomes from Plants and Archaea were included in the search pipeline; but no candidates satisfying the combined validation criteria (TM-score ≥ 0.5 and conservation of the six-cysteine disulfide scaffold) were identified. To quantify these distributional patterns, we calculated Phyletic Spread (Si, number of unique phyla/kingdoms) and Phyletic Depth (Di, average gene copies per species) for each lineage, stratified by taxonomic supergroup (Fig. 2). Within Arthropoda, both lineages are restricted to a single phylum (Si = 1) but diverge markedly in copy number: the Conventional lineage shows a higher depth (Di = 2.4) compared to the Ancestral form (Di = 1.3), reflecting lineage-specific gene duplication within this clade. Outside Arthropoda, the Ancestral lineage displays the broadest taxonomic distribution (Si = 8, Di = 2.4), spanning basal metazoans, Lophotrochozoa, Fungi, and Bacteria, while Conventional domains outside Arthropoda show more restricted spread (Si = 6, Di = 1.2). Together, these metrics confirm the broader phylogenetic distribution of the Ancestral architecture and the arthropod-specific expansion of the Conventional lineage.

To evaluate whether the identified bacterial PLD-encoding loci represent genuine bacterial sequences rather than artifacts of eukaryotic database contamination, we performed a local genomic context analysis and assembly quality assessment for all 24 bacterial accessions (23 source genomes; Supplementary Fig. 1, Supplementary Table 1). The local genomic context analysis of the ± 5 flanking genes surrounding each PLD locus revealed a total of 195 flanking genes across all 24 loci, none of which carried eukaryotic-specific functional annotations. All detected flanking gene functions are consistent with bacterial genomic contexts, including RNA polymerase subunits (*rpoB*, *rpoC*), 30S ribosomal proteins (*rpsL*), chaperone systems (DnaK/DnaJ/GrpE), endolytic transglycosylases (MltG), LuxR-family transcriptional regulators, and hypothetical proteins. Assembly quality assessment confirmed that all PLD-containing genomes display genome sizes, GC contents, and assembly characteristics consistent with their respective taxonomic groups: Myxobacteria-associated hits reside in large, GC-rich genomes (10.8–13.0 Mb; 68.5–71.5% GC), two of which are complete closed genomes, while CPR-associated hits derive from compact MAGs (509–1,500 kb; median 880 kb; GC 35.0–49.5%) consistent with the known biology of this clade. Together, these results are inconsistent with an eukaryotic contamination, which — if present — would be expected to introduce flanking genes with eukaryotic annotations at or near the Pacifastin locus^26^ and support the interpretation that the identified PLD homologues are genuine bacterial sequences, although experimental validation is required to reach a definitive conclusion (see Limitations).

### Lineage-specific sequence signatures

**Fig. 3:**
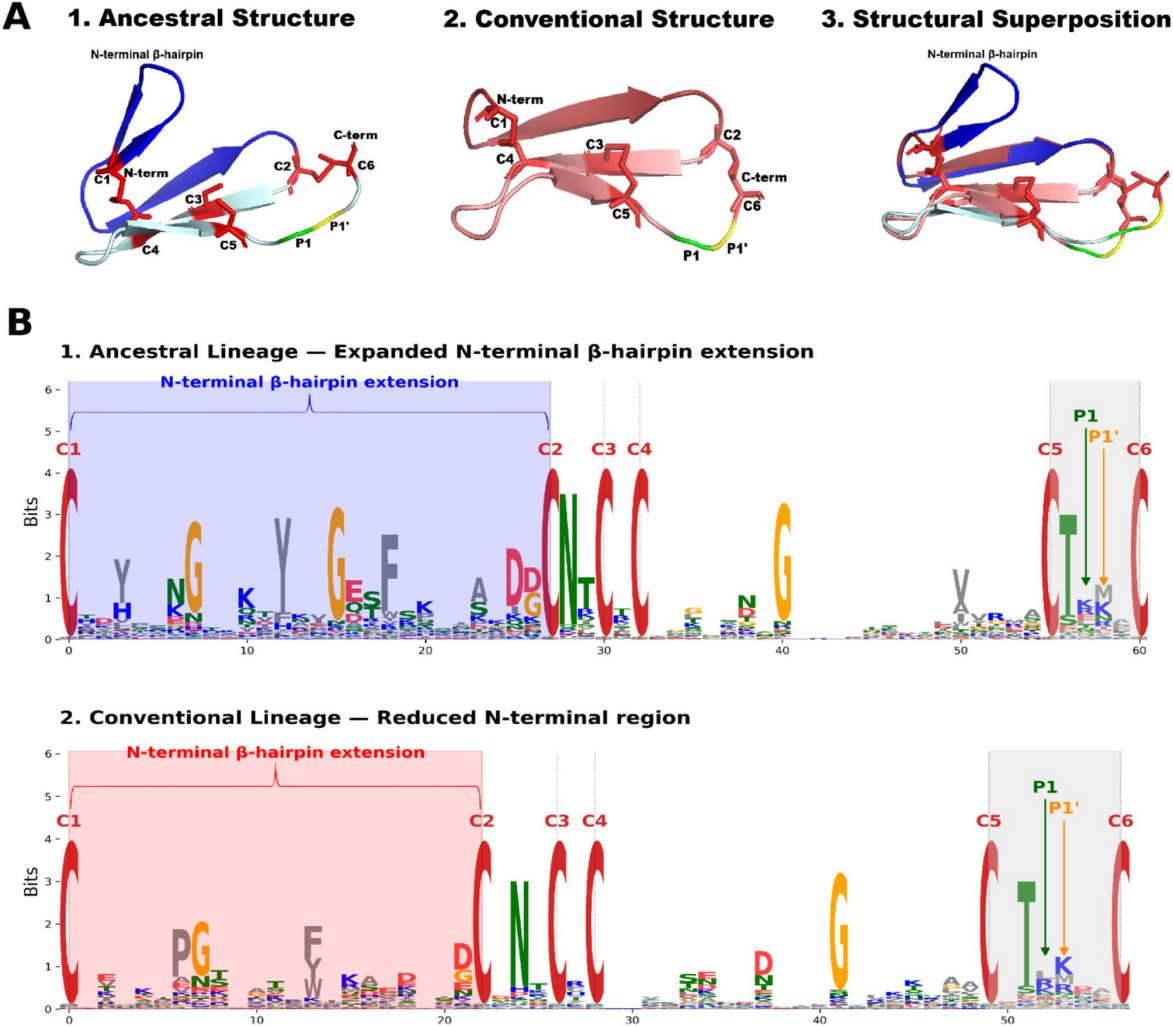
Structural signatures of Pacifastin lineage divergence. **(A)** Three-dimensional architectural comparison. Representative structural models of the Ancestral (1) and Conventional (2) lineages. The N-terminal region is highlighted in blue (Ancestral, extended β-hairpin conformation) and red (Conventional, compact conformation). The superposition (3) demonstrates the strict spatial conservation of the disulfide-bonded core (red sticks), while highlighting the conformational divergence of the N-terminal region. The reactive site residues P1 (green) and P1’ (yellow) are marked in both structural models, indicating their position within the Cys5-Xaa-P1-P1’-Xaa-Cys6 loop. Cysteines C1-C6 are shown in red. **(B)** Structure-anchored sequence logos derived from the multiple sequence alignment of validated domains. Arrows above each logo delimit the N-terminal β-hairpin extension region. The Ancestral lineage (1) is characterized by an expanded N-terminal β-hairpin extension (blue background), enriched in Glycine (G) and aromatic residues (Y, F). The Conventional lineage (2) displays a reduced N-terminal region (red background) frequently stabilized by a conserved Proline (P) residue. Key conserved features include the six-cysteine scaffold (C1–C6, red labels), an invariant Asparagine (N) forming the rigid C2-N-C3 motif, and a structural Glycine (G) in the C4-C5 loop. The reactive site positions P1 (green arrow) and P1’ (yellow arrow) are marked in both logos, indicating their location within the Cys5-Cys6 loop (gray shaded region).

To dissect the molecular basis of the architectural divergence, we performed a structural superposition of statistically representative models from both lineages. As illustrated in Fig. 3A, the core disulfide scaffold maintained a rigid spatial conservation, whereas the N-terminal region exhibited an extensive conformational deviation. To quantify these features, we generated structure-anchored sequence logos derived from the multiple sequence alignment of the validated domains (Fig. 3B). The six-cysteine scaffold (C1 to C6) served as the strictly conserved backbone of the superfamily. Beyond this disulfide topology, we identified two universal key motifs essential for the Pacifastin fold: an invariant Asparagine residue immediately following Cys2, forming a rigid C-N–C core likely stabilized by hydrogen bonding, and a highly conserved Glycine residue within the C4-C5 loop, which functions as a structural hinge to orient the reactive site loop.

The variable N-terminal β-hairpin extension exhibited distinct physicochemical signatures that correlate with the observed architectural bifurcation. The Ancestral lineage was characterized by a major expansion in this N-terminal extension (spanning positions ~ 1–27), which was enriched in Aromatic residues (Tyr/Phe) and features a characteristic insertion of an extra Glycine residue. This composition suggests a conformationally dynamic region capable of flexible interactions, consistent with a role in macromolecular complexes. In contrast, the Conventional lineage displayed a reduced N-terminal region (positions ~ 1–22), lacking the β-hairpin extension, frequently stabilized by a conserved Proline residue, providing the structural rigidity required for a compact, soluble inhibitor (Fig. 3B).

### Phylogenetic distribution of structural divergence between ancestral and conventional lineages

**Fig. 4:**
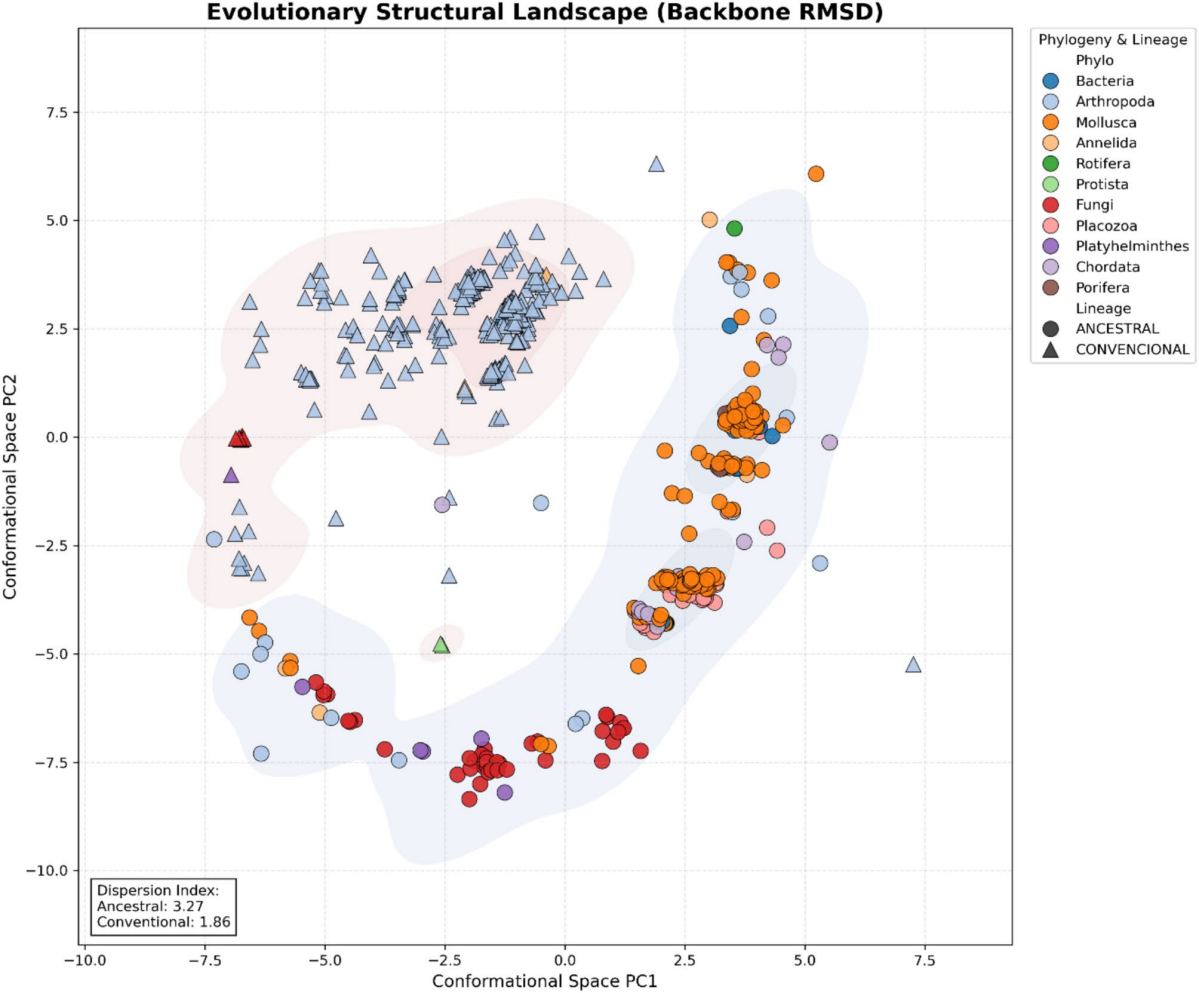
Evolutionary structural landscape of Pacifastin domains. Multidimensional Scaling (MDS) plot derived from an all-vs-all backbone RMSD alignment of representative structural models. The background shading estimates the density of the conformational space occupied by the diffuse Ancestral lineage (Blue, Dispersion Index 3.27) and the compact Conventional lineage (Red, Dispersion Index 1.86). Markers are shaped by lineage (Circle: Ancestral; Triangle: Conventional) and colored by phylogenetic group. The Conventional space dominated by Arthropods, indicates a lineage-specific structural divergence. In contrast, the Ancestral space contains a diverse mix of all phylogenetic groups, including ancestral-type Arthropods dispersed among Mollusca and Fungi, reflects a plastic evolutionary origin. Distinct sub-clusters are visible within the ancestral cloud, with Mollusk-rich aggregations in the right quadrant and Fungi-rich regions in the lower quadrant near the structural boundary.

To determine whether the architectural divergence defined by the N-terminal β-hairpin extension affects the global protein fold stability and diversity, we performed a Multidimensional Scaling (MDS) analysis of the backbone RMSD. The structural landscape analysis revealed a dichotomy that correlates with phylogenetic history. The Conventional lineage formed a dense, highly compact cluster (Dispersion Index 1.86) located primarily in the upper-left quadrant (Fig. 4). This conformational subspace was dominated almost exclusively by Arthropods. Notably, this cluster contained only rare sporadic representatives from other groups, including a small cluster of four Fungi, one Platyhelminth, and one Annelida sequences.

In contrast, the Ancestral lineage exhibited a high degree of structural heterogeneity (Dispersion Index 3.27), occupying a broad and diffuse conformational space (Fig. 4, blue background) that encompassed representatives from all evaluated phylogenetic groups. The internal architecture of this ancestral cloud revealed distinct phylogenetic substructures. The right quadrant featured dense conglomerations dominated by Mollusca mixed with other metazoans, including Placozoa. As the distribution extended towards the lower quadrant, approaching the Conventional boundary, the clustering became less dense and featured aggregations of Fungi mixed with dispersed sequences from other groups. Arthropod sequences belonging to the ancestral lineage were scattered throughout this entire cloud, co-localizing with Mollusks and other basal metazoans.

### Ancestral and conventional lineages differ in their residue-level non-covalent contact networks

**Fig. 5:**
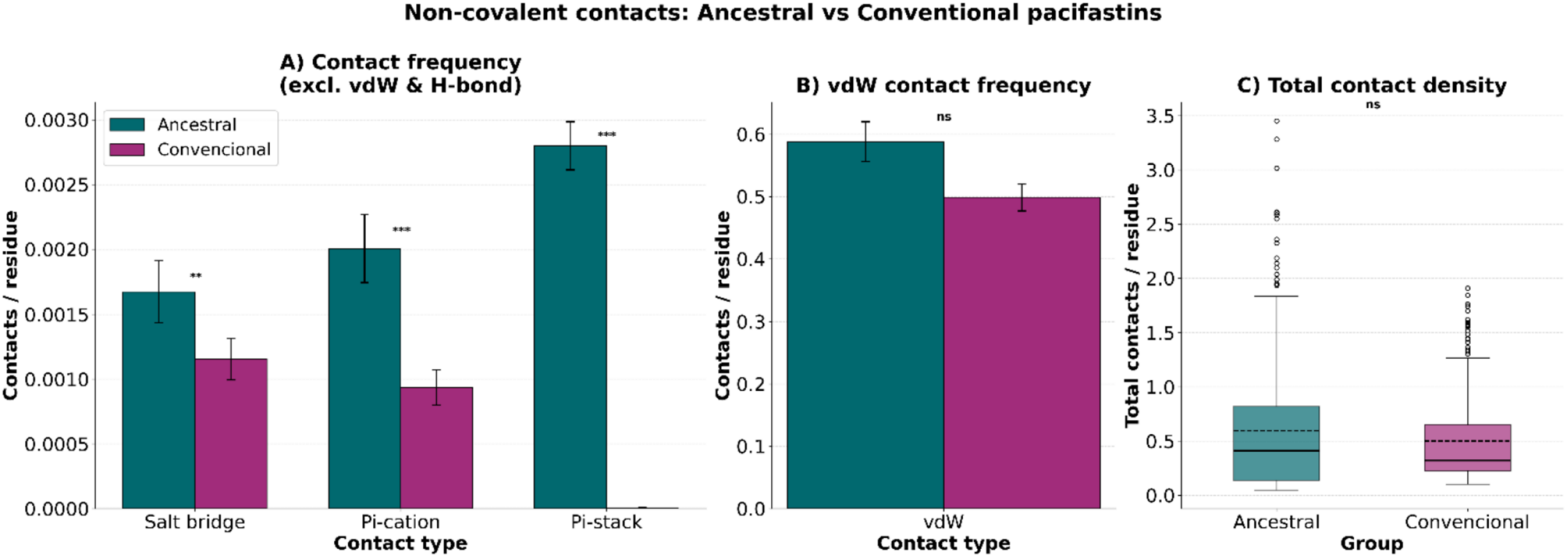
Non-covalent contact profile of Ancestral and Conventional pacifastin-like domains. **(A)** Mean frequency of salt bridge, pi-cation, and pi-stacking contacts per residue. **(B)** Mean frequency of van der Waals (vdW) contacts per residue, shown separately due to the difference in scale relative to panel A. **(C)** Total contact density distribution per protein. Statistical comparisons were performed using two-sided Mann–Whitney U tests. Error bars represent Standard Error of the Mean. **p < 0.01, ***p < 0.001, ns = not significant. H-bond contacts were not detected in the analyzed structures and are therefore not shown.

To determine whether the architectural divergence between Ancestral and Conventional pacifastin-like domains is accompanied by differences in residue-level contact networks, we performed a systematic non-covalent contact analysis. Ancestral domains showed significantly higher frequencies of salt bridge (Ancestral: 0.00167 ± 0.00024 contacts/residue [95% CI: 0.00121–0.00214]; Conventional: 0.00115 ± 0.00016 [95% CI: 0.00084–0.00147]), pi-cation (Ancestral: 0.00201 ± 0.00026 [95% CI: 0.00149–0.00252]; Conventional: 0.00094 ± 0.00014 [95% CI: 0.00067–0.00120]), and pi-stacking contacts (Ancestral: 0.00280 ± 0.00019 [95% CI: 0.00244–0.00317]; Conventional: 0.000004 ± 0.000004 [95% CI: ~ 0.00]) per residue compared to Conventional domains (Fig. 5, Panel A). Van der Waals contacts dominated the contact profiles of both lineages (Ancestral: 0.5881 ± 0.0320 [95% CI: 0.5254–0.6508]; Conventional: 0.4987 ± 0.0219 [95% CI: 0.4557–0.5417]) and were not significantly different between groups (p > 0.05, Fig. 5, Panel B). Accordingly, the total contact density per residue did not differ significantly between lineages (Ancestral: 0.5946 ± 0.0323 SEM [95% CI: 0.5313–0.6579]; Conventional: 0.5008 ± 0.0220 [95% CI: 0.4577–0.5439]; p = 0.74, Fig. 5, Panel C). Collectively, these results indicate that while the two lineages share a similar overall contact density, they differ markedly in the qualitative composition of their non-covalent interaction networks, with the Ancestral lineage exhibiting a contact signature enriched in electrostatic and aromatic interactions.

### Functional diversification and phylogenetic distribution of the P1-P1’ active site

**Fig. 6:**
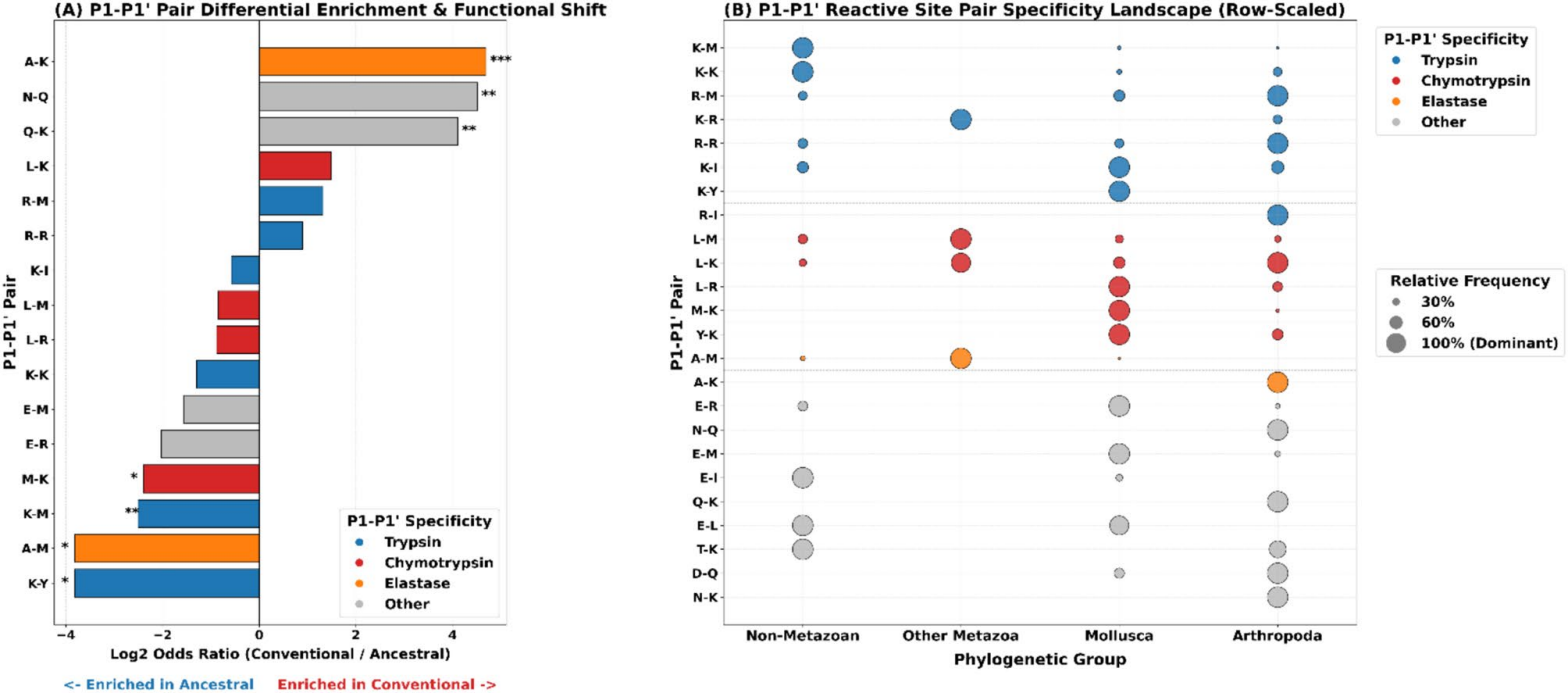
Evolutionary remodeling and phylogenomic specialization of the P1 – P1’ active site. **(A)** Differential enrichment plot (Log2 Odds Ratio) contrasting P1 residue frequency between Conventional and Ancestral lineages. Bars extending right indicate significant enrichment in the Conventional lineage. Bars extending left indicate significant retention in the Ancestral lineage. Asterisks denote statistical significance (Fisher’s Exact Test: *** *p* < 0.001, ** *p* < 0.01, * *p* < 0.05). **(B)** Functional dot plot showing the relative dominance of residues across phylogenetic groups (individual row-scaled: the group with maximum usage for a given amino acid is set to dot size 1.0). Colors indicate predicted specificity (Blue: Trypsin; Red: Chymotrypsin; Orange: Elastase; Gray: Other). The ancestral state (Mollusca, Non-Metazoan) is defined by maximum dominance in Lysine (K) and a distinctive signature of Glutamic Acid (E) and Threonine (T). The Arthropoda lineage shows a functional inversion, dominated by Arginine (R) over Lysine, and maximally expanded into Leucine (L), Phenylalanine (F), and Alanine (A).

We investigated whether the transition to the Conventional lineage involved a shift in protease specificity by analyzing the differential enrichment and phylogenomic dominance of P1-P1’ pairs in the active site. Within the structure-anchored alignment, the P1 residue occupies the second position of the Cys5-Cys6 loop (Cys5-Xaa-P1-P1’-Xaa-Cys6), as explicitly marked in Figs. 3 and 5. The analysis revealed a complex pattern of functional remodeling characterized by both biochemical optimization and adaptive radiation (Fig. 6).

The differential enrichment analysis indicated a lineage-specific divergence in reactive site pair composition (Fig. 6A). A total of 16 P1-P1’ pairs had sufficient statistical power for analysis, of which seven showed significant differential enrichment between lineages (Fisher’s Exact Test, p < 0.05). The Ancestral lineage exhibited a unique specificity profile significantly enriched in Trypsin-like pairs carrying hydrophobic residues, specifically K-Y (p = 0.010), A-M (p = 0.010), and K-M (p = 0.003), as well as the Chymotrypsin-like pair M–K (p = 0.047).In contrast, the Conventional lineage displayed significant enrichment in three pairs: A-K (Log2OR = 4.69, p < 0.001), N-Q (Log2OR = 4.52, p = 0.001), and Q-K (Log2OR = 4.11, p = 0.007). This pattern reflects a coordinated shift at both the P1 and P1’s positions.

The phylogenomic landscape (Fig. 6B) revealed the distribution of 80 unique P1-P1’ pairs across the four major taxonomic groups, showing that the ancestral state depicted a distinct combinatorial profile where Mollusca and Non-Metazoan groups exhibited maximum relative dominance in Trypsin-like pairs (K-M, K-K, R-M), while also showing dominant usage of pairs carrying non-canonical P1 residues such as Glutamic Acid (E-R, E-M) and Glutamine (Q-K).

The Arthropod lineage diverts from this ancestral pattern through two major combinatorial shifts evident in the plot. First, arthropods show a conservative refinement in Trypsin-like pairs, from the ancestral K-M dominance toward R-M— a substitution that fine-tunes binding affinity within the same mechanistic inhibitory class, as both Lys and Arg engage the S1 specificity pocket of trypsin-like serine proteases through equivalent electrostatic interactions. Second, Arthropoda shows exclusive or maximum relative dominance in the Elastase-like pair A-K (Log2OR = 4.69, p < 0.001), — the functionally transformative innovation of the Conventional lineage, as it represents a qualitative shift in target protease class from trypsin-like to elastase-like specificity, substantially broadening the inhibitory spectrum beyond what a conservative Lys-to-Arg substitution achieves.

### Architectural simplification and network topology revealed a transition from multi-domain scaffolds to solitary inhibitory units

**Fig. 7:**
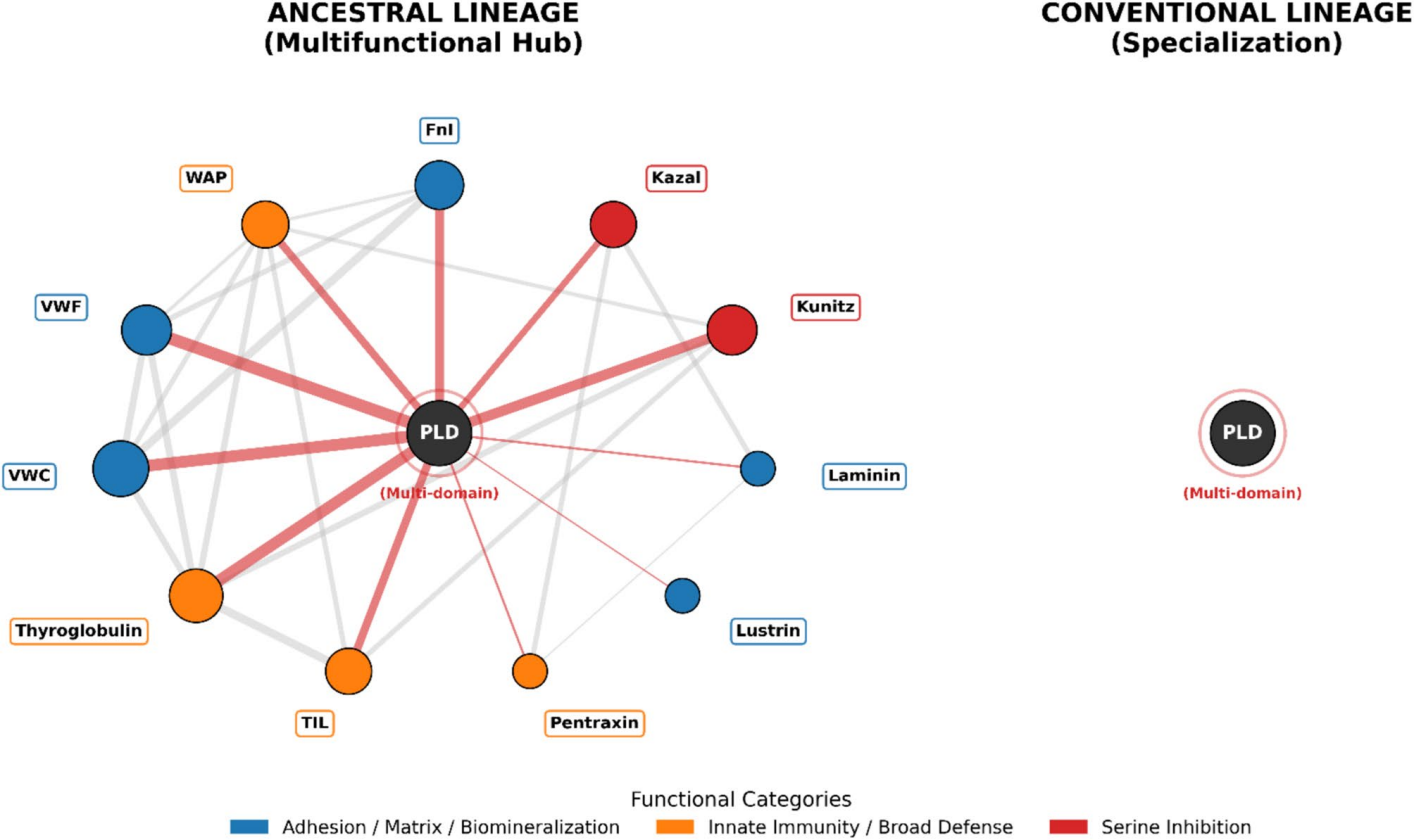
Functional network topology of multi-domain Ancestral scaffolds and solitary Conventional PLDs. Co-occurrence networks of Pfam domains are visualized for the two architectural pacifastin groups, nodes are colored according to Gene Ontology (GO) functional classification. **(A)** The Ancestral group displays a high complexity “Hub” architecture where the central Pacifastin domain (PLD) is an anchor for diverse functional modules. Blue nodes represent domains associated with cell adhesion, extracellular matrix organization, and biomineralization (e.g., Laminin, Lustrin, VWC, FnI). Orange nodes represent domains involved in pattern recognition and broad-spectrum innate immunity (e.g., Pentraxin, TIL, WAP, Thyroglobulin). Red nodes indicate auxiliary serine protease inhibitors (Kazal, Kunitz). **(B)** The Conventional group exhibits extreme evolutionary simplification, appearing as a functionally isolated node. In both panels, red edges denote direct structural association with the Pacifastin core, gray edges represent peripheral functional crosstalk among accessory domains, and the external ring around the central node indicates the presence of homologous tandem repeats.

To determine if the structural divergence between Conventional and Ancestral pacifastins correlates with distinct architectural contexts beyond the diversification of the P1-P1’ active site, we performed a comparative domain architecture analysis integrated with network topology visualization (Fig. 7). This combined approach revealed a dichotomy in modular complexity: whereas Ancestral domains are predominantly embedded within multi-domain architectures, Conventional domains occur almost exclusively as standalone units.

The Ancestral lineage (n = 239) displayed a high complexity “Multifunctional Hub” topology. While 67.4% of proteins were solitary, a substantial fraction (32.6%, n = 78) formed multi-domain architectures physically linked to diverse functional clusters. The network highlighted a strong association with “Adhesion” domains (Laminin G, Lustrin, and Von Willebrand factor type C).

The Conventional lineage (n = 155) exhibited extreme evolutionary simplification, appearing as a functionally isolated node. We observed a strict specialization towards solitary architecture, with 100% of the proteins consisting exclusively of PLDs.

### Evolutionary switch towards proteolytic processing as a derived arthropod innovation

**Fig. 8:**
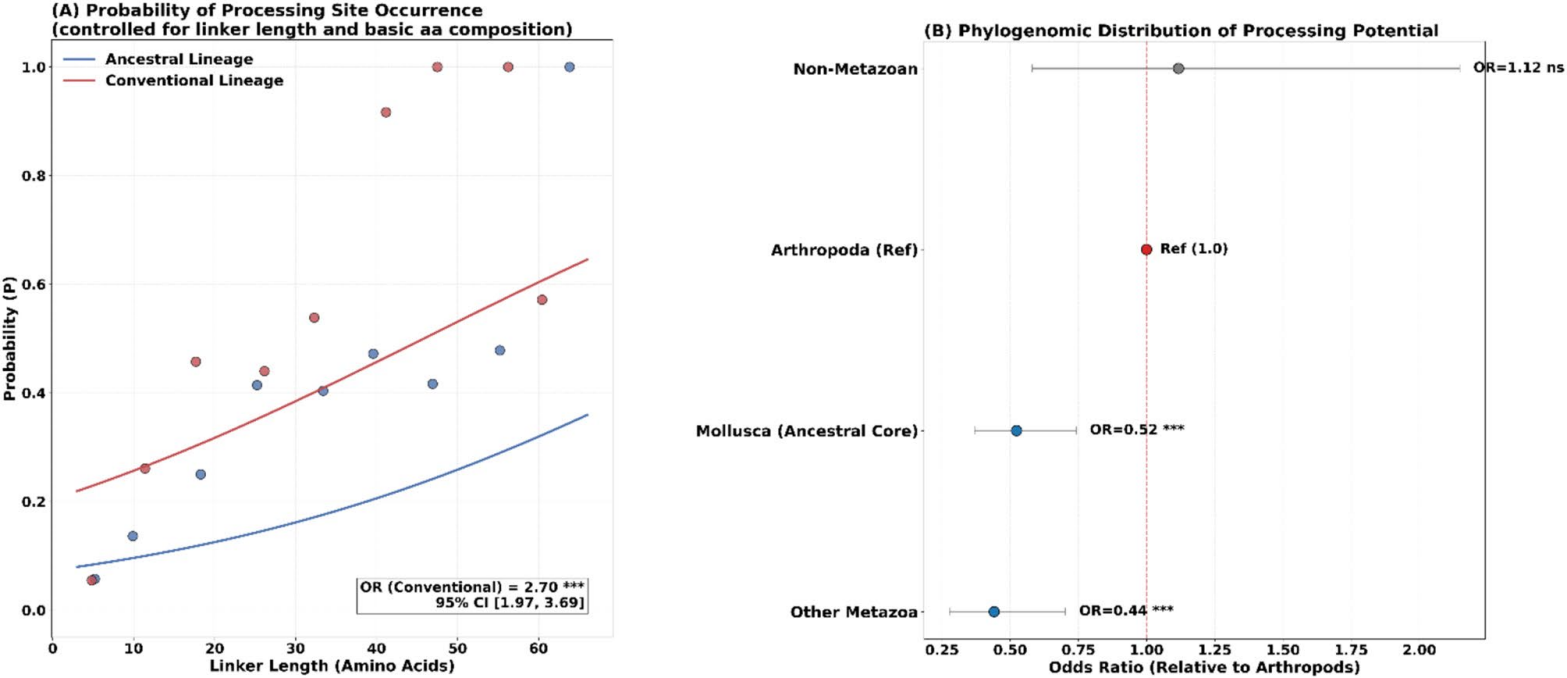
Possibility of finding a canonical cleavage motif according to architectural and taxonomic groups. **(A)** Logistic regression curves modeling the possibility of finding a canonical cleavage motif (KR/RR/RK/KK) as a function of inter-domain linker length (number of amino acid residues between consecutive Pacifastin-like domains). The Conventional lineage (red) shows a significantly higher possibility trend than the Ancestral lineage (blue) across the entire length range (*OR* = 2.24,* p* < 1.0e^-7^). Points represent binned averages of observed data. **(B)** Forest plot of Odds Ratios (*OR*) derived from a phylogenomic GLM, with Arthropoda set as the reference (dashed line, *OR* = 1.0). Ancestral groups, including Mollusca (blue) and Other Metazoa (gray, pooled ancestral lineages), exhibit significantly lower.

We investigated whether the proposed structural transition to the Conventional lineage was accompanied by a shift in post-translational processing mechanisms, specifically the ability to release single-domain effectors from a multi-domain precursor. The initial analysis revealed that inter-domain linkers — defined as the amino acid sequences separating two consecutive Pacifastin-like domains within the same multi-domain precursor — in the Conventional lineage were significantly enriched in dibasic cleavage motifs compared to the Ancestral lineage. To determine if this was driven by active selection rather than length variation or basic amino acid composition, we modeled the probability of cleavage using an extended logistic regression model controlling for linker length and the fraction of basic residues (Fig. 8A). The model demonstrated a robust functional divergence: for any given linker length, the Conventional lineage exhibited a consistently higher probability of harboring cleavage sites. The length-adjusted Odds Ratio indicated that Conventional linkers were 2.7 times more likely to be processed than Ancestral linkers (OR = 2.70, 95% CI [1.97, 3.70], p = 6.58 × 10⁻^1^⁰). This demonstrated that the accumulation of cleavage sites was a non-stochastic feature of the Conventional lineage, consistent with a transition from stable “scaffold” proteins to cleavable precursors.

To determine if this high-processing phenotype is a general trend in higher Metazoa or a lineage-specific innovation, we stratified the GLM analysis by taxonomic group, setting Arthropoda as the statistical reference (*OR* = 1.0) (Fig. 8B). The analysis revealed that the high proteolytic potential was unique to Arthropods. The Mollusca lineage, which represented the primary reservoir of ancestral diversity, exhibited a significantly lower processing potential (*OR* = 0.52, *p* < 0.001), indicating that their linkers are evolutionarily constrained to remain stable. On the other hand, the consolidated “Other Metazoa” group (comprising Cnidaria, Annelida, and Platyhelminthes) exhibited an even lower processing potential (*OR* = 0.44, p < 0.001), placing them significantly to the left of the reference line.

## Discussion

The Pacifastin family has been widely regarded as an arthropod-specific innovation, with functional roles largely restricted to the regulation of crustacean and insect innate immunity^15,19^. Our structural phylogenomic analysis fundamentally challenged this view and substantially expands upon the only prior large-scale study of the family, which identified 83 precursor proteins with 284 PLDs across 55 species from 3 Metazoan phyla using sequence-based methods, with non-arthropod representation limited to a single precursor in Placozoa (42 PLDs) and one in Onychophora (4 PLDs). The present study identified 394 unique proteins across 11 high-level taxonomic groups — a ~ 4.7-fold increase in protein coverage — and revealed entire phyla (Rotifera, Cnidaria, Fungi, Bacteria) completely absent from previous sequence-based surveys. Nearly 30% of this expanded dataset (n = 118 proteins) was undetectable by the HMM-based approach, underscoring that the apparent restriction of the family to Arthropoda was a methodological artifact rather than a biological reality. We demonstrated that the canonical six-cysteine scaffold is an ancient innovation that predates the Ecdysozoan radiation, with cryptic homologues present in deep-branching lineages including Cnidaria, Rotifera, and Fungi.

The failure of previous studies to identify these homologues highlights the limitations of standard homology searching tools and the bias inherent in publicly available reference models (e.g., Pfam), which were constructed predominantly from the canonical short-loop architecture originally described in arthropods. These limitations hinder the detection of divergent variants, particularly in small, disulfide-rich proteins (SDRPs). As observed in other SDRP families, the rigid disulfide framework acts as an evolutionary buffer, allowing the inter-cysteine loops to undergo rapid sequence divergence without compromising the global fold^20,21^. This phenomenon of "structural persistence amidst sequence erosion" explains the discrepancy observed in detection sensitivity (Fig. 1), where nearly 30% of our dataset—including entire phyla like Rotifera—remained undetectable by HMMER but was readily detected by Foldseek.

The identification of structural homologs across such vast phylogenetic distances raises the fundamental question of their evolutionary origin. While we acknowledge that convergent evolution towards a stable fold is a theoretical possibility—particularly in small, disulfide-rich proteins where the folding landscape is constrained—the structural evidence strongly favors a model of divergent evolution. It is a fundamental principle of molecular evolution that protein structure is conserved over far longer timescales than sequence^27^. Furthermore, the Pacifastin fold is defined not merely by its compact size, but by a topologically complex six-cysteine scaffold with a specific, interlaced disulfide connectivity (Cys1–Cys4, Cys2–Cys6, Cys3–Cys5). The probability of independently recreating this specific three-disulfide register through convergence is constrained by the limited sequence space compatible with correct disulfide pairing and by the structural role of the Cys3–Cys5 bridge, which braces the reactive loop geometry — an additional combinatorial barrier that makes repeated independent invention unlikely. Accordingly, our findings suggest that the Pacifastin lineage is not an arthropod innovation, but an ancient molecular scaffold whose evolutionary roots extend at minimum to the base of the Metazoan radiation and plausibly, pending experimental confirmation, to even earlier stages in the diversification of life.

Most intriguingly, our structural screen identified a significant population of PLD homologues in Bacteria (n = 23, Structure-only). Prior to any evolutionary interpretation, we note that the genomic context analysis and assembly quality assessment presented in this study (Supplementary Fig. 1, Supplementary Table 1) are inconsistent with eukaryotic contamination as the source of these sequences: zero eukaryotic-signal genes were detected among the 195 flanking genes analyzed, and all source genomes display assembly properties consistent with their respective bacterial lineages (Myxobacteria and CPR; see Methods). Nonetheless, experimental validation remains necessary, and the following interpretations should be regarded as working hypotheses. While convergent evolution towards a stable disulfide-rich fold is possible, the strict conservation of the topological cysteine spacing is consistent with a distinct evolutionary link rather than independent convergence. This distribution pattern—presence in Bacteria, Fungi, and Metazoa, but absence in Plants or Archaea—is consistent with at least two non-mutually exclusive hypotheses: ancient Horizontal Gene Transfer (HGT) events, a phenomenon frequently observed in secretion-related virulence factors^28^; or, alternatively, a deep common ancestry predating the bacteria–eukaryote divergence, in which case these bacterial sequences may represent structural vestiges of a primordial inhibitory scaffold that was co-opted and elaborated in the eukaryotic lineage. We consider the latter hypothesis particularly speculative and note that it requires direct experimental support before stronger conclusions can be drawn.

A scrambled cysteine-matched decoy control was not incorporated into the pipeline, as the mandatory topological curation step operates but *Functional diversification and Phylogenetic* a more stringent and independent specificity filter than a TM-score-based FDR estimation. Critically, these two approaches operate on orthogonal criteria: while a decoy control estimates false positives based on global structural similarity — the same space captured by the TM-score — the topological verification of the disulfide bonding pattern (Cys1-Cys4, Cys2-Cys6, Cys3-Cys5) is performed directly from atomic coordinates, independently of both ESMFold and Foldseek metrics. This independent filter also resolves the circularity of AI-prediction pipelines^29^, as topological verification constitutes a third, independent line of evidence beyond prediction and structural alignment.

Despite this deep ancestry, the “Conventional” architecture remains uniquely restricted to Arthropods. Phyletic Spread and Depth analysis (Fig. 2) revealed that this lineage underwent a specific burst expansion (gene duplication) within this phylum (*D*_*i*_ = *2.4*), contrasting with the low copy number of the ancestral form (*D*_*i*_ = *1.3*), consistent with a burst of gene duplication events following the architectural transition. This pattern is further supported by our functional network analysis (Fig. 7), which suggests that the shift toward the conventional architecture may have been associated with a transition from "matrix-embedded regulation” to “systemic” defense.

The Ancestral lineage, dominant in basal metazoans and Lophotrochozoans, is characterized by a “Multifunctional Hub” topology. The fact that 32.6% of these proteins form multi-domain architectures physically linked to adhesion modules (Laminin G, Lustrin, VWC) and biomineralization domains suggests that ancestral pacifastins were not freely circulating inhibitors but they were anchored to the extracellular matrix (ECM) or basement membranes^30–32^. In this context, the N-terminal β-hairpin extension enriched in Glycine (Fig. 3B) characteristic of the Ancestral architecture likely functioned as a flexible hinge, facilitating protein–protein interactions within bulky macromolecular complexes. The extended and flexible nature of this β-hairpin — in contrast to the compact, Proline-stabilized N-terminal region of the Conventional lineage — suggests a direct structural basis for the higher interaction potential observed in ancestral forms.

The emergence of the Conventional lineage in Arthropods coincides with a loss of these adhesive anchors —resulting in a 100% solitary domain architecture— and the concomitant acquisition of proteolytic processing sites in the inter-domain linkers. We propose a “Liberation” model, where the evolution of protease-sensitive linkers is consistent with a mechanism that may have enabled arthropods to release single-domain inhibitors from large precursors^12^. This model is statistically underpinned by our GLM analysis, which demonstrated that inter-domain linkers in the Conventional lineage are 2.7 times more likely (OR = 2.7, 95% CI [1.97, 3.70], p = 6.58 × 10⁻^1^⁰, adjusted for linker length and basic amino acid composition) to harbor cleavage sites than their ancestral counterparts. This association is consistent with the hypothesis that the acquisition of protease-sensitive linkers facilitated the transition from a static, matrix-bound regulator toward a diffusible, high-mobility effector capable of rapidly dispersing through the open hemolymph system to neutralize systemic threats. In addition, we cannot rule out that these accessible inter-domain linkers might serve a dual function, acting as ‘bait regions’ that recruit target proteases to facilitate their subsequent inhibition by the adjacent PLD, a mechanism analogous to other protease trap inhibitors^33^.

The transition to a soluble state is associated with strict biophysical constraints, consistent with a process of structural convergence mediated by specific amino acid selection. This phenomenon was quantitatively captured by our conformational landscape analysis (Fig. 4), which revealed that the Conventional lineage occupies a tightly constrained subspace (Dispersion Index 1.86) in sharp contrast to the structurally heterogeneous Ancestral cloud (Dispersion Index 3.27). It is important to note that the Dispersion Index quantifies structural diversity across the lineage as a whole, not the thermodynamic stability of individual domains — a lower index indicates that members of the Conventional lineage have converged toward a more homogeneous structural solution, consistent with but not demonstrative of increased individual domain stability^34^. This convergence is consistent with a selection-driven constraint process in which selection constrained the accessible conformational space, but confirmation of enhanced thermodynamic stability would require molecular dynamics simulations or experimental thermal denaturation assays on representative domains from each lineage. While the global six-cysteine scaffold remains invariant^35^, the variable loops exhibit lineage-specific adaptations to manage the trade-off between flexibility and stability. The Ancestral lineage features an extended N-terminal β-hairpin enriched in Glycine and aromatic residues (Fig. 3B). Structurally, Glycine acts as a "conformational hinge," imparting the high flexibility required for induced-fit interactions^36^. This supports our hypothesis that ancestral PLDs functioned within large macromolecular complexes (e.g., ECM or cell receptors), where loop plasticity is advantageous for binding diverse partners. In contrast, the Conventional lineage underwent a rigidification process. The loss of the N-terminal β-hairpin extension is accompanied by the fixation of a conserved Proline residue (Fig. 3B). Proline is unique among amino acids in restricting the backbone dihedral angles (Φ), thereby reducing the entropy of the unfolded state and stabilizing the folded protein^37^. This "Proline-lock" predictably to constrain backbone conformational freedom, consistent with a more rigid structural chassis — a biophysical consequence that would be advantageous for a small, soluble inhibitor functioning within a protease-rich hemolymph. The reduced backbone entropy associated with Proline fixation has been well documented in model peptide systems and is consistent with enhanced folded-state stability^38^. However, direct experimental validation via thermal shift assays or molecular dynamics simulations would be required to confirm the predicted increase in thermodynamic stability.

The non-covalent contact analysis provided direct quantitative support for this architectural divergence (Fig. 5). The significantly higher frequencies of salt bridge, pi-cation, and pi-stacking contacts observed in Ancestral domains are consistent with the enrichment of aromatic (Tyrosine, Phenylalanine) and charged (Glutamic acid) residues in the N-terminal β-hairpin extension of this lineage. Importantly, these contact types are not indicative of structural rigidity but rather of surface-mediated intermolecular recognition. Salt bridges are dynamic electrostatic interactions that form and break depending on the local environment and binding partner^39,40^, while pi-cation and pi-stacking interactions are characteristically present *Within the structure-anchored alignment,* at protein–protein interfaces, where they mediate flexible recognition of diverse molecular partners^41,42^. This contact signature supports the hypothesis that ancestral PLDs functioned within large macromolecular complexes anchored to the extracellular matrix, where surface plasticity is advantageous for engaging multiple binding partners. In contrast, the reduction of these contact types in the Conventional lineage, combined with the Proline-lock that physically restricts backbone dihedral angles, is consistent with the transition toward a compact, internally rigid domain optimized for soluble inhibitory function in the hemolymph. The conservation of van der Waals contacts across both lineages further supports the notion that this interaction type provides a structural baseline required for the integrity of the six-cysteine scaffold, independent of the architectural class.

Having established a stable, soluble scaffold, the Arthropod lineage channeled its evolutionary potential into the diversification of the reactive site. Our P1-P1’ pair differential enrichment analysis (Fig. 6) showed that the Ancestral active site, is dominated by Trypsin-like pairs carrying hydrophobic P1’ residues (K-Y, K-M, A-M), suggesting a primordial function involving broad electrostatic interactions^43^ or the inhibition of endogenous trypsin-like proteases involved in tissue remodeling or developmental signaling^44^. In contrast, the Conventional lineage displays a clear signature of adaptive radiation driven by functional diversification^45^. The enrichment of Arginine-containing pairs (R-M) (p < 0.001) represents an optimization of anti-trypsin affinity, essential for regulating complex physiological processes ranging from digestion and embryonic development to the prophenoloxidase activating system^46^. The significant enrichment of the Elastase-like pair A-K and the Chymotrypsin-associated pair L-K could result in broadening of the inhibitory spectrum to target chymotrypsin and elastase, consistent with roles in hematophagy and metabolic regulation^47^. Notably, the coordinated selection at both P1 and P1’ positions — as evidenced by the enrichment of specific pairs rather than individual residues — suggests that reactive site diversification in arthropods involved combinatorial optimization of the inhibitory interface, consistent with the known role of the P1’ residue in fine-tuning protease selectivity^48^. This implies that the Pacifastin P1-P1’ sites diversified to target a broad-spectrum defense against invasive proteolysis. This “dual strategy”, conserving a rigid, Proline-stabilized scaffold while hyper-variable the P1-P1’ sites, allowed arthropods to repurpose an ancient regulatory domain into a versatile component of systemic physiology.

In conclusion, this study elucidates the comprehensive evolutionary history of the Pacifastin family, establishing it not as an arthropod-specific innovation but as a fundamental metazoan lineage. We define a major evolutionary transition from an ancestral “Cellular Scaffold” phenotype—characterized by conformational flexibility and matrix retention—to a derived “Soluble Effector” phenotype, optimized for systemic dispersion. This functional shift is consistent with two synergistic molecular mechanisms supported by statistical associations in our data: the statistically significant acquisition of proteolytic processing motifs associated with release from the extracellular matrix, and the structural convergence of the fold consistent with the biophysical requirements of thermodynamic stability in the hemolymph. Ultimately, these findings underscore that the limits of sequence-based detection do not define the boundaries of protein families; rather, deep evolutionary relationships remain accessible through the rigorous application of structural phylogenomics.

While our structural phylogenomic framework provides compelling evidence for the deep ancestry of Pacifastins, several limitations must be acknowledged. Although AlphaFold2 and ESMFold yield high-confidence models, they remain computational predictions; while the combination of high pLDDT scores, stringent TM-score thresholds, and mandatory topological curation provides strong evidence for the authenticity of structure-only homologues, experimental structural validation via X-ray crystallography or NMR is desirable for the highly divergent sequence-undetectable homologues identified. The bacterial PLD-like homologues warrant particular caution: although zero eukaryotic-signal genes were detected among the 195 flanking genes analyzed and all source genomes display assembly properties consistent with their respective bacterial lineages, definitive exclusion of metagenomic contamination requires experimental validation (e.g., PCR confirmation on independent genomic DNA preparations). All evolutionary interpretations regarding these loci — including proposed HGT events and deep common ancestry — should therefore be regarded as working hypotheses pending experimental support. Also, while the strict conservation of the cysteine scaffold and active site geometry strongly implies functional preservation, the protease inhibitory activity of these non-metazoan homologues — including the bacterial variants — has not yet been validated in vitro. Finally, our proposed “Liberation” model and the transition from matrix-tethered to soluble effectors are inferred from statistically robust genomic patterns; however, the temporal ordering of this transition — whether architectural simplification preceded or followed the acquisition of proteolytic cleavage sites — cannot be resolved from cross-sectional genomic data alone, and an alternative scenario in which cleavage site acquisition drove subsequent architectural simplification remains equally consistent with the observed patterns. Future cell biological studies tracking the processing and localization of ancestral variants are necessary to substantiate this proposed evolutionary trajectory.

## Materials and methods

### Pacifastin homologues detection via iterative profile-based searching

To comprehensively identify members of the pacifastin serine protease inhibitor family, a sensitive iterative search strategy was employed. Sequence similarity searches were performed primarily using JackHMMER (of the HMMER package v3.3^23^), against the NCBI non-redundant protein (nr) and nr50 (clustered at 50% identity) UniProtKB database.

The iterative search was initiated using a seed derived from the canonical, biochemically characterized pacifastin protein from *P. leniusculus*. To isolate the conserved functional domain, this full-length protein sequence was first queried against the Pfam and NCBI Conserved Domain Database (CDD) repositories using hmmscan^49^. The resulting HMM profile (Hidden Markov Model) corresponding to the core Pacifastin-like domain (PLD) was extracted and employed as the seed profile for the first search iteration.

Multiple rounds of iterative searching were executed. Significant homologues (E-value < 1e-10) recovered in each round were manually inspected to verify the conservation of the canonical six-cysteine architecture (Cys1-Cys6) of the pacifastin domain. Sequences that passed this curation filter were used to generate updated profiles for the subsequent iteration. To manage redundancy and define intermediate homologous groups during the iterative process, the retrieved sequence sets were clustered using MMseqs^50^.

Finally, a consolidated HMM profile was constructed from the high-confidence multiple sequence alignment (MSA) of the curated dataset. This final profile was used to perform exhaustive searches (hmmsearch) against expanded databases, including the NCBI genome and metagenome collections^51^, to identify homologues in taxonomic phyla where pacifastin presence had not been previously reported or was presumed absent.

### Retrieval of coding sequences (CDS)

The iterative searches yielded lists of homologous protein identifiers, including UniProtKB accession numbers. To retrieve the corresponding nucleotide coding sequences (CDS), UniProtKB accession numbers were mapped to the GenBank (CDS) or EMBL (CDS) database using the UniProt Retrieve/ID Mapping web service^52^, and the resulting nucleotide sequences were batch-downloaded using the NCBI E-utilities command-line tool efetch^53^.

### Structural modeling and structure-based homology search

To investigate the structural diversity of the pacifastin family and identify cryptic homologues undetectable by sequence-based methods, a high-throughput structural analysis workflow was implemented. First, the retrieved nucleotide coding sequences were translated into amino acid sequences. To optimize computational resources and prevent clustering biases arising from large, non-catalytic accessory domains (e.g., Kazal or von Willebrand factor domains), a domain-centric clustering strategy was applied. The specific coordinates of Pacifastin-like domains (PLDs) were identified in all sequences using hmmscan against the Pacifastin_I profile (PF05375). These PLD subsequences were computationally extracted.

Given that standard high-speed clustering methods (e.g., MMseqs2) proved ineffective for grouping these short (approx. 30–40 aa) and divergent domain sequences, a high-sensitivity protocol was implemented using CD-HIT^54^. Sequences were clustered at a 50% identity threshold (-c 0.5) using a word size of 2 (-n 2), a parameter combination optimized for finding homology between short, divergent sequences, and header descriptions were preserved (-d 0). The resulting representative PLD sequences were then mapped back to their parent proteins (using seqkit grep)^55^ to establish a non-redundant dataset of full-length pacifastin precursors.

Three-dimensional structures for these representative full-length proteins were predicted using ColabFold, an optimized implementation of the AlphaFold2 algorithm^56^. Modeling the full-length precursors was prioritized over isolated domains to preserve the native structural context and stability of the domains. Subsequently, the atomic coordinates corresponding exclusively to the PLD regions were computationally excised from the generated full-length PDB models, utilizing the domain boundaries defined by the HMMER analysis^57^.

To assess whether predicting PLDs within a full-length multidomain context introduces conformational bias, we performed a systematic structural comparison between PLD structures excised from full-length ESMFold predictions and the corresponding domain-only ESMFold predictions for a representative set of 50 domains (25 Ancestral, 25 Conventional). For each domain, the PLD amino acid sequence delimited by HMMER domain coordinates was extracted and submitted independently to ESMFold. The resulting domain-only structure was superimposed onto the corresponding domain excised from the full-length model using Cα-based structural alignment, and the backbone RMSD was computed. The 50 pairwise comparisons yielded extremely low RMSD values across both lineages: overall mean RMSD = 0.014 ± 0.053 Å (range 0.002–0.381 Å; n = 50). Lineage-specific values were: Ancestral mean 0.023 ± 0.075 Å (median 0.004 Å); Conventional mean 0.005 ± 0.006 Å (median 0.003 Å). For reference, RMSD values below 0.5 Å are considered indicative of essentially identical conformations in Cα-based structural comparisons^58^. These results confirm that domain structures excised from full-length ESMFold models are structurally equivalent to domain-only predictions, and that the multidomain prediction context does not introduce a systematic conformational bias in the PLD fold.

These isolated PLD structures served as queries for sensitive structure-versus-structure searches against the AlphaFold Protein Structure Database and the Protein Data Bank (PDB) using Foldseek^25^. This approach facilitated the detection of distant evolutionary relatives sharing the characteristic Pacifastin fold despite low sequence identity. Foldseek searches were performed using a TM-score acceptance threshold of 0.5 (default parameter). The quality of the AlphaFold2/ColabFold structural predictions was assessed using per-residue pLDDT scores (reported in Supplementary File 1). For the 189 query structures used in Foldseek searches, the global mean pLDDT was 87.15, with 94.7% of structures achieving mean pLDDT ≥ 70. For the complete dataset of 676 predicted Pacifastin-like domains, the mean pLDDT was 83.26, with 94.8% achieving mean pLDDT ≥ 70. Structural similarities were further validated and quantified using DALIlite, generating structural alignments and Z-scores to assess the statistical significance of the matches.

### Comparative set analysis and validation of cryptic homologues

To distinguish between sequence-identifiable and cryptic homologues, a comparative analysis was performed between the dataset obtained via HMM-profile search (sequence-based) and the dataset retrieved via Foldseek (structure-based). The identifiers were normalized, and the datasets were segregated into three categories as illustrated in Fig. 1A: (1) Common set (detected by both methods), (2) Sequence-unique (detected only by HMM), and (3) Structure-unique (detected only by Foldseek). The "Structure-unique" candidates, representing potential cryptic homologues with lost sequence identity, underwent a manual curation process based on structural topology. The extracted domain subsequences were inspected to verify the topological conservation of the disulfide-bonded scaffold (Cys1-Cys6), rejecting any false positives lacking the essential cysteine core. This mandatory manual curation step served as an explicit specificity filter: any structure-unique candidate lacking the canonical Cys1-Cys6 topological spacing with the bonding pattern Cys1-Cys4, Cys2-Cys6, Cys3-Cys5 was rejected, regardless of TM-score".

### Evolutionary metrics and stratified analysis

To quantify the evolutionary modes of Pacifastin lineages, taxonomic information (Organism and Phylum/Kingdom) was retrieved for all validated sequences using a hybrid approach of NCBI Entrez API querying based on accession numbers and local text mining of FASTA headers for non-standard identifiers. Taxonomic assignments were manually curated to resolve ambiguities.

Following the framework proposed by *Burroughs *et al*.*^59^, we calculated two metrics: Phyletic Spread (*S*_*i*_) and Phyletic Depth (*D*_*i*_). *S*_*i*_ is defined as the count of unique high-level taxonomic groups (phyla or kingdoms) in which a lineage is present, representing evolutionary dispersion. *D*_*i*_ is calculated as the total number of sequences in a lineage divided by the total number of unique species detected, representing the average gene copy number per genome (proxy for gene expansion/duplication).

To address sampling bias and resolve distinct evolutionary histories, the dataset was stratified into “Arthropoda” and "Non-Arthropoda" supergroups. Metrics were calculated independently for each stratum and lineage combination and visualized as a scatter plot to define evolutionary landscapes.

### Architectural classification of PLD loops

During the curation process, different architectural variations were observed in the N-terminal region connecting the first two cysteines (Cys1-Cys2). To systematically classify these variations, a motif-based filtering strategy was implemented using custom Python scripts. Proteins were categorized into two architectural classes: “Conventional”, exhibiting the canonical short loop (9–12 residues) typical of characterized pacifastins, and “Ancestral” (non-conventional structure, designated as such based on the phylogenomic evidence presented herein), defined by an N-terminal β-hairpin extension of 13 to 20 residues. This classification was mapped against the three homology sets to assess correlations between structural divergence and detection method sensitivity, as quantified in Fig. 1B.

### PLD structural modeling, sequence analysis and motif visualization

To visualize the PLDs architectural differences without bias, representative models for the Ancestral and Conventional lineages were selected based on statistical centrality. We calculated the median length of the N-terminal β-hairpin extension for each dataset. The protein exhibiting the loop length closest to the group median and the shortest overall sequence length (to minimize terminal disorder) was selected as the structural prototype. Three-dimensional structures were predicted using the ESMFold API^60^. Structural superposition was performed in PyMOL using the conserved six-cysteine core as the anchor reference (RMSD minimization on Cys Cα atoms).

To characterize lineage-specific sequence motifs without the alignment artifacts introduced by standard global alignment algorithms on hypervariable loops, we implemented a structure-anchored alignment strategy. Domain subsequences were computationally extracted using regular expressions anchored to the six conserved cysteine residues (C1 to C6). The variable loops between cysteines were aligned independently to preserve residue composition patterns. Sequence logos were generated using the Python library logomaker^61^, calculating the information content (bits) for each position relative to the background amino acid frequency.

### Active site specificity and functional diversification analysis

To evaluate the functional diversification of the Pacifastin family, we analyzed the identity of the P1-P1’s residues within the canonical reactive loop (Cys_5_-Xaa-P1-P1’-Xaa-Cys_6_). P1-P1’ residues were extracted from all validated domains and functionally classified based on their predicted protease inhibition profile: Trypsin-like (Arg, Lys), Chymotrypsin-like (Leu, Phe, Tyr, Met, Trp), Elastase-like (Ala, Val, Gly), and others^16^.

To quantify evolutionary bias in residue selection, we performed a differential enrichment analysis using the Log_2_ Odds Ratio (Log_2_OR) metric:

$${Log}_{2}(OR)={log}_{2}\left(\frac{{Frequency}_{Conventional}}{{Frequency}_{Ancestral}}\right)$$

Positive values indicate enrichment in the Conventional lineage, while negative values indicate a higher frequency in the Ancestral lineage. Statistical significance was assessed using Fisher’s Exact Test with a correction for low counts. This approach allows us to distinguish between stochastic variation and active lineage-specific selection.

To further resolve these lineage-specific preferences independent of overall amino acid abundance and sample size imbalances across diverse taxonomic groups, we generated a Functional Dot Plot with Row-Scaling normalization^62^. For each P1-P1’ pairs, the phylogenetic group with the highest raw frequency was assigned to a relative dominance value of 1.0 (represented by the maximum dot size). Other groups were scaled proportionally relative to that maximum. This approach highlights which lineage effectively specialized in the usage of a specific P1-P1’ pair, distinguishing between ubiquitous P1-P1’ pairs and those showing genuine lineage-specific dominance.

### Structural modeling and conformational landscape analysis

To assess the evolution on the PLD structural topology, we performed a comprehensive conformational landscape analysis. To define the structural boundaries of each lineage, the validated domain sequences were first clustered using CD-HIT with a 70% sequence identity threshold. This process yielded a non-redundant dataset comprising 352 Ancestral and 324 Conventional representative sequences. Three-dimensional models for all representatives were generated using ESMFold (Evolutionary Scale Modeling), a high-accuracy language model for structure prediction that estimates backbone geometry without requiring homologous templates^60^.

We constructed a global structural similarity matrix by calculating the pairwise Root Mean Square Deviation (RMSD) of the alpha-carbon backbone between all structures (all-vs-all comparison). To visualize this high-dimensional dataset, the distance matrix was projected into a two-dimensional conformational space using Metric Multidimensional Scaling (MDS)^60^. The dimensions in this projection (Coordinate 1 and Coordinate 2) represent synthetic spatial axes optimized to preserve pairwise distances; thus, the Euclidean distance between any two points in the plot serves as a direct proxy for their structural dissimilarity (RMSD in Angstroms). To map the evolutionary trajectory onto this landscape, we overlaid phylogenetic metadata onto the structural coordinates, distinguishing taxonomic groups by color and major lineages by marker shape. Finally, to quantify the degree of structural constraint versus plasticity, we calculated the Structural Dispersion Index for each lineage, defined as the mean Euclidean distance of all constituent structures to their respective geometric centroid within the MDS space.

### Residue-level non-covalent contact analysis

To assess whether the structural divergence between Ancestral and Conventional pacifastin-like domains is reflected in differences in their non-covalent contact networks, we performed a static contact analysis using GetContacts v1.0 on the non-redundant structural dataset previously described (352 Ancestral and 324 Conventional representative models, obtained by CD-HIT clustering at 70% sequence identity). The following interaction types were computed: salt bridges (sb), pi-cation interactions (pc), pi-stacking (ps), and van der Waals contacts (vdw). Contact frequencies were normalized by the number of residues of each domain to account for differences in domain length. Group comparisons were performed using two-sided Mann–Whitney U tests. Statistical significance was set at p < 0.05.

### Domain architecture annotation

To formally annotate the complete domain architecture of all retrieved protein sequences, the full curated set was scanned against the complete Pfam-A HMM database using hmmscan. A two-tiered analytical strategy was then applied to the results. The primary objective was to confirm the identity and boundaries of the target domain; therefore, the presence, coordinates, and statistical significance of the core Pacifastin_I domain (PF05375) were verified for all sequences, which served to validate the inclusion of each sequence in the final dataset.

A second objective was to identify all co-occurring or “accessory” domains, which are critical for characterizing the functional diversification of the family, such as the Kazal, VWF, and TIL domains. For this architectural analysis, all hits corresponding to functional families other than Pacifastin domain (PF05375) were considered significant and retained only if they met a standard domain inclusion E-value threshold of < 1e-5.

### Comparative analysis of modular architecture

To quantify the architectural divergence between the identified lineages, the final dataset was stratified into two groups: the “Conventional” lineage (n = 155), comprising proteins with the canonical short-loop topology, and the “Ancestral” lineage (n = 239), comprising proteins validated with the extended-loop architecture. Both datasets were subjected to a comprehensive domain annotation using hmmscan against the Pfam-A database with a significance threshold of E-value < 1e-5.

A custom Python pipeline was developed to classify the modular organization of each protein based on the hmmscan output. Domains identified as Pacifastin (PF05375) were designated as “core” domains. Proteins were classified into two architectural categories: (1) "PLD-only", defined as proteins containing exclusively PLDs (single or multiple repeats) or those lacking any detectable Pfam domains despite structural validation (pure cryptic variants); and (2) “Complex”, defined as proteins containing at least one non-Pacifastin accessory domain (e.g., VWC, Kunitz, TIL). The ratio of PLD-only to Complex architecture was calculated for both lineages to assess the degree of evolutionary specialization.

### Functional network topology construction

To visualize the organization of functional modules within the Pacifastin superfamily, we constructed co-occurrence networks for both the Ancestral and Conventional lineages. The hmmscan outputs were parsed to generate an adjacency matrix where nodes represented distinct Pfam domains and edges represented their co-occurrence within the same protein architecture. To provide biological context, we implemented a Gene Ontology (GO)-based classification scheme^63^. Accessory domains were mapped to functional categories.

Network visualization was performed using the Python libraries networkx and matplotlib^62^. We employed a radial layout algorithm to arrange accessory domains around the central Pacifastin core to facilitate the comparison of hub connectivity. Visual parameters were mapped to statistical properties: node size was scaled logarithmically to the domain frequency, and edge width was scaled to the co-occurrence count. Edges connecting accessory domains directly to the Pacifastin core were highlighted to distinguish them from peripheral crosstalk interactions between accessory modules.

### Proteolytic processing prediction

To reconstruct the evolutionary history of post-translational processing in the Pacifastin family, we analyzed the inter-domain linker regions of all multi-domain precursors. Inter-domain linkers were defined as the amino acid sequences located between two consecutive Pacifastin-like domains (PLDs) within the same multi-domain precursor protein, bounded by the C-terminus of one PLD and the N-terminus of the immediately next PLD as determined by the HMMER domain coordinate annotations. Linker length was defined as the number of amino acid residues comprising this inter-domain interval. Linker sequences were computationally extracted based on validated PLD coordinates. Linkers longer than 50 amino acids were excluded to remove potential structural loops or disordered domains not relevant for canonical processing. A pattern recognition search was performed to identify canonical dibasic cleavage motifs (KR, RR, RK, KK) recognized by proprotein convertases^15^.

Simple frequency analysis of cleavage sites is potentially confounded by sequence length, as longer linkers have a higher stochastic probability of harboring motifs. To control this variable, we implemented a Generalized Linear Model (GLM) with a binomial distribution (Logistic Regression). The model was defined by the following equation:

$$logit\left(P\right)=\beta 0+\beta 1\left(Length\right)+\beta k\left(Group\right)$$

In this model, $logit\left(P\right)$ represents the probability of a linker containing at least one cleavage site; $\beta 0$ denotes the intercept; $\beta 1$ is the regression coefficient for length, which controls for the stochastic accumulation of motifs in longer sequences; and $\beta k$ represents the coefficient for the taxonomic group (the predictor of interest).

We performed two complementary analyses:

- Group 1. Lineage Modeling: Comparing the Conventional versus Ancestral lineages to assess the global evolutionary trend.
- Group 2. Phylogenomic Stratification: Comparing functional taxonomic groups—Arthropoda (Reference), Mollusca (Ancestral Core), Other Metazoa (pooled ancestral lineages), and non-Metazoa—to determine the origin of the phenotype. Odds Ratios (OR) were calculated relative to the Arthropod baseline (OR = 1.0).

### Genomic context and assembly quality analysis

To assess whether the bacterial putative Pacifastin-like domain (PLD)-encoding loci are embedded in genuine bacterial genomic contexts, we performed a systematic local genomic context analysis for all 24 bacterial accessions identified in this study. GFF3 annotation files were downloaded from NCBI for each source genome using the Entrez API (Biopython v1.81). For each accession, the PLD-encoding gene was identified by its protein accession number within the GFF3 feature table, and the five immediately upstream and downstream annotated CDS features (± 5 gene window) were extracted based on genomic coordinates within the same contig. Each flanking gene was assigned to one of eight functional categories based on its “product” attribute: (1) Transcription/Translation, (2) Transport/Membrane, (3) Cell wall/Carbohydrate metabolism, (4) Phosphorylation/Signaling, (5) Regulation, (6) Metabolism/Biosynthesis, (7) Hypothetical/Uncharacterized, and (8) Eukaryotic signal. The Eukaryotic signal category was assigned to genes annotated with any of the following functions: histones, tubulins, actins, spliceosomal components, 40S or 60S ribosomal subunit proteins, and ubiquitin. Assembly statistics (genome size, number of scaffolds, scaffold N50, GC content, genome coverage, and assembly level) were retrieved from NCBI Assembly for all 23 source genomes via manual inspection of NCBI Assembly records. Organisms were classified into two taxonomic groups — Myxobacteria (Pseudomonadati; complete or high-quality draft genomes) and Candidate Phyla Radiation (CPR; metagenome-assembled genomes) — based on their NCBI taxonomy.

## Supplementary Information

Supplementary Information 1.

Supplementary Information 2.

## Data Availability

All data underlying this article are available in Zenodo and GitHub. The complete dataset of predicted structural models (PDB format) and the curated Multiple Sequence Alignments are deposited in Zenodo at doi.org/10.5281/zenodo.18616634. The custom Python pipelines developed for the iterative HMM-based search, architectural loop classification, and Generalized Linear Model (GLM) analysis are available on GitHub at [https://github.com/Daniel-Rdgz/Pacifastin_Structural_Phylogenomics] (https:/github.com/Daniel-Rdgz/Pacifastin_Structural_Phylogenomics).
