# Supplementary material for: Structure-guided analysis of divergent homologs unveils deep ancestry and arthropod specialization of the pacifastin family
Source: Sci Rep. 2026 May 20;16:22875. doi: 10.1038/s41598-026-52748-5 (PMC13388995; doi:10.1038/s41598-026-52748-5)

Supplementary table 1. Assembly quality for bacterial source genomes

| **accession** | **organism** | **assembly_name** | **assembly_level** | **genome_size_kb** | **scaffold_N50_kb** | **scaffold_L50** | **contig_N50_kb** | **contig_L50** | **gc_percent** |
| --- | --- | --- | --- | --- | --- | --- | --- | --- | --- |
| AKT37495.1 | Chondromyces crocatus | ASM118929v1 | Complete Genome | 11400 | 11400 | 1 | 11400 | 1 | 68.5 |
| AKT37496.1 | Chondromyces crocatus | ASM118929v1 | Complete Genome | 11400 | 11400 | 1 | 11400 | 1 | 68.5 |
| CAN94267.1 | Sorangium cellulosum So ce56 | ASM6716v1 | Complete Genome | 13000 | 13000 | 1 | 13000 | 1 | 71.5 |
| KKQ86892.1 | Candidatus Woesebacteria bacterium GW2011_GWB1_38_8b | ASM99311v1 | Contig | 903.9 | 32.9 | 9 | 32.9 | 9 | 37.5 |
| KKR99052.1 | Candidatus Uhrbacteria bacterium GW2011_GWC1_41_20 | ASM99676v1 | Contig | 915.3 | 40.2 | 9 | 40.2 | 9 | 41 |
| KXK08942.1 | candidate division WS6 bacterium OLB21 | ASM156730v1 | Contig | 1100 | 71.2 | 6 | 71.2 | 6 | 38.5 |
| KXK26912.1 | candidate division WS6 bacterium OLB20 | ASM156735v1 | Contig | 1500 | 744.7 | 1 | 744.7 | 1 | 49.5 |
| KYF71355.1 | Sorangium cellulosum | sce1107.1 | Scaffold | 12000 | 5.4 | 640 | 5.4 | 643 | 70.5 |
| MRG92455.1 | Polyangium spumosum | ASM964984v1 | Scaffold | 10800 | 821.6 | 6 | 712.5 | 7 | 69.5 |
| OGC49363.1 | candidate division WWE3 bacterium RIFCSPLOWO2_01_FULL_37_15 | ASM177289v1 | Scaffold | 509.1 | 10.7 | 17 | 8.6 | 18 | 37 |
| OGC53823.1 | candidate division WWE3 bacterium RIFCSPHIGHO2_02_FULL_38_14 | ASM177286v1 | Scaffold | 941.1 | 94.7 | 4 | 47.9 | 7 | 37 |
| OGD03279.1 | Candidatus Amesbacteria bacterium RIFCSPLOWO2_01_FULL_48_25 | ASM177369v1 | Scaffold | 938.8 | 164.8 | 3 | 138.7 | 3 | 47.5 |
| OGK39131.1 | Candidatus Roizmanbacteria bacterium RIFCSPLOWO2_01_FULL_35_13 | ASM178963v1 | Scaffold | 1500 | 33.8 | 13 | 26.1 | 16 | 35.5 |
| OGK42414.1 | Candidatus Roizmanbacteria bacterium RIFCSPLOWO2_01_FULL_37_12 | ASM178967v1 | Scaffold | 880.5 | 65.1 | 5 | 37.7 | 9 | 37 |
| OGK44663.1 | Candidatus Roizmanbacteria bacterium RIFCSPLOWO2_01_FULL_37_16 | ASM178969v1 | Scaffold | 1100 | 26.6 | 14 | 18.3 | 16 | 36 |
| OGM65121.1 | Candidatus Woesebacteria bacterium RIFCSPLOWO2_01_FULL_39_25 | ASM179310v1 | Scaffold | 1300 | 144.9 | 3 | 141.7 | 3 | 37.5 |
| OIP87587.1 | Candidatus Shapirobacteria bacterium CG2_30_35_20 | ASM187366v1 | Scaffold | 568 | 10.2 | 17 | 10 | 18 | 35.5 |
| PIV07531.1 | Candidatus Shapirobacteria bacterium CG03_land_8_20_14_0_80_35_14 | ASM277992v1 | Scaffold | 640 | 7 | 30 | 6.2 | 34 | 35 |
| PIX67905.1 | Candidatus Shapirobacteria bacterium CG_4_10_14_3_um_filter_35_13 | ASM278294v1 | Scaffold | 540.3 | 7.6 | 26 | 7 | 28 | 35.5 |
| PIZ60880.1 | Candidatus Shapirobacteria bacterium CG_4_10_14_0_2_um_filter_40_12 | ASM279221v1 | Scaffold | 781.5 | 12.9 | 20 | 12.6 | 21 | 40 |
| PIZ68592.1 | Candidatus Roizmanbacteria bacterium CG_4_10_14_0_2_um_filter_36_35 | ASM279235v1 | Scaffold | 850.7 | 10.8 | 25 | 10.2 | 26 | 35.5 |
| PJA51202.1 | Candidatus Shapirobacteria bacterium CG_4_9_14_3_um_filter_36_12 | ASM279117v1 | Scaffold | 543.3 | 8.7 | 23 | 7.5 | 26 | 35.5 |
| PJC79727.1 | Candidatus Shapirobacteria bacterium CG_4_8_14_3_um_filter_35_11 | ASM278799v1 | Scaffold | 739.4 | 16.8 | 13 | 9.4 | 20 | 39.5 |
| PJE66709.1 | Candidatus Shapirobacteria bacterium CG10_big_fil_rev_8_21_14_0_10_36_6 | ASM279371v1 | Scaffold | 583.1 | 3.2 | 56 | 2.2 | 81 | 36 |

Supplementary figure 1. Genomic context of bacterial putative pacifastin-encoding loci.


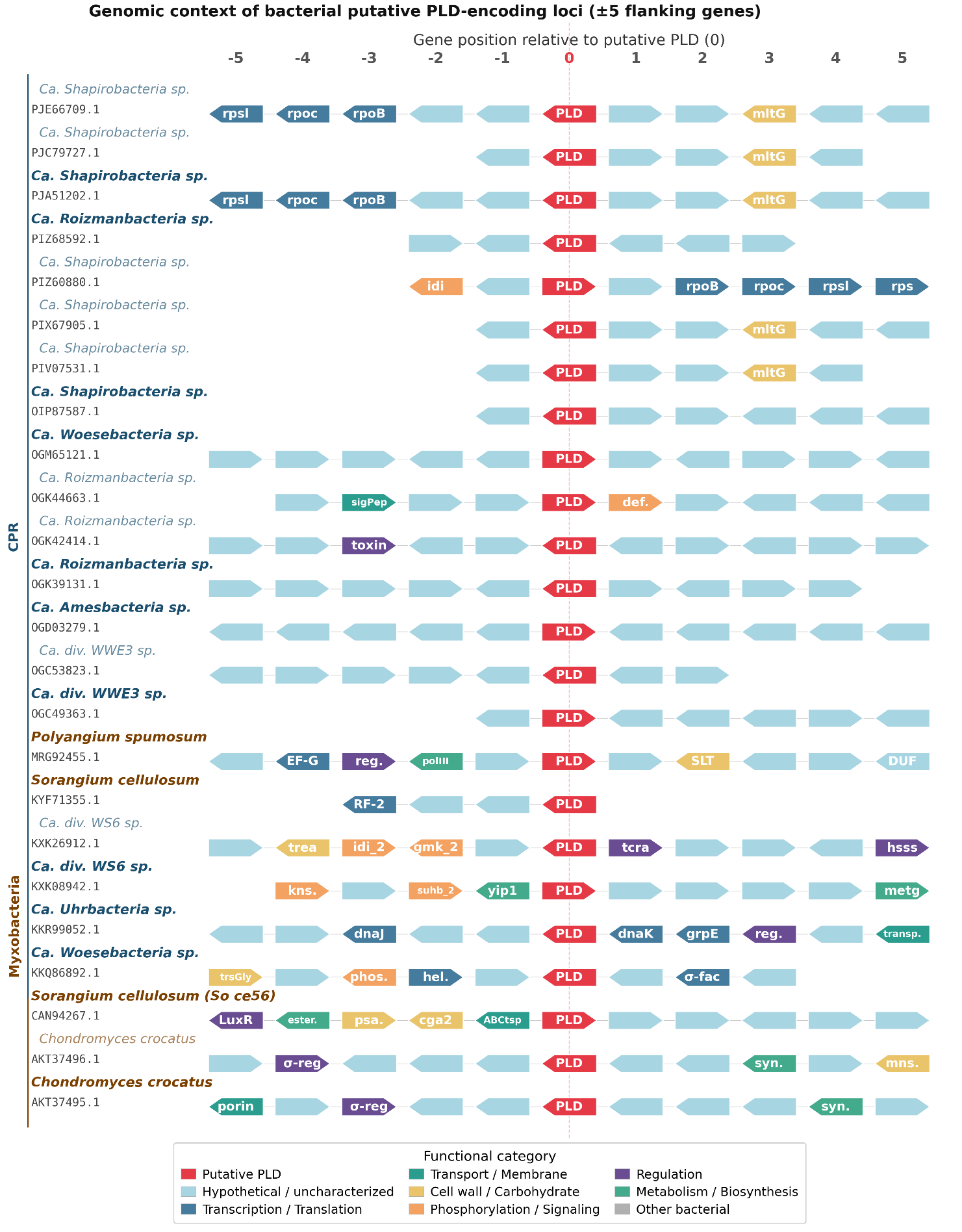

Supplement: Supplementary file 2 — Supplementary Information 2. [file 41598_2026_52748_MOESM2_ESM.docx]
